# Coiled-coil register transitions and coupling with the effector’s inhibitory site enables high fold changes in blue light–regulated diguanylate cyclases

**DOI:** 10.1016/j.jbc.2025.111020

**Published:** 2025-12-06

**Authors:** Uršula Vide, Gabriela Shickle, Julia Schwekendiek, Andreas Winkler

**Affiliations:** 1 Institute of Biochemistry, Graz University of Technology, Graz, Austria; 2 BioTechMed-Graz, Graz, Austria

**Keywords:** photoreceptor, cyclase, signal integration, hydrogen deuterium exchange mass spectrometry, kinetics, screening

## Abstract

Cellular signaling cascades rely on transfer of information from one protein to another or within a single protein. To facilitate signal integration, specific structural motifs evolved that allow signal processing and also enable modular downstream response integration, facilitating sophisticated regulatory mechanisms. On a structural level, especially coiled-coil helices are frequently observed as signaling motifs. In diguanylate cyclases (DGCs) featuring GGDEF domains, N-terminal coiled-coils frequently activate systems by rearrangements of the interdimer active site. The variety of sensory domains that modulate this structural equilibrium in response to different stimuli highlights the importance of DGCs in bacterial adaptation. One interesting example of sensor DGCs is blue light–activated light–oxygen–voltage (LOV)–GGDEF couples. Here, we describe molecular details of a two-stage mechanism that allows tight dark-state inhibition while enabling high enzymatic activities upon illumination, achieving fold changes exceeding 10,000-fold. Using an *in vivo* activity assay, we screened amino acid substitutions at the inhibitory interface and the sensor-effector linker region to identify variants that promote enzymatic activity in the dark. In combination with chimeras of LOV and GGDEF domains preventing inhibitory interface formation, we successfully stabilized elongated active-state conformations and confirmed the role of the inhibitory interface between sensor and effector in the tight dark-state inhibition. Interestingly, the initially generated chimeras are still light regulatable as long as the linker sequence is not stabilized in either inhibiting or stimulating coiled-coil register. Our results offer valuable insights for potential optogenetic applications but also demonstrate inherent challenges associated with *Methylotenera* sp. LOV-activated DGCs.

Cellular signaling is fundamental for organisms to respond to their environment and to maintain homeostasis (1). A central aspect of cellular signaling involves the use of characteristic structural motifs that mediate interactions, allow modifications, and/or facilitate the transmission of signals within and between proteins (2, 3). Employing recurring structural motifs like coiled coils—also sometimes termed signaling helices in this context—allows a more efficient evolvability than specific domain interfaces for each sensor–effector pair (4).

Coiled coils are structural motifs characterized by two (or more) alpha-helices twisted around each other in a supercoiled fashion. Parallel dimeric coiled coils are typically defined by a heptad repeat pattern, denoted as (*abcdefg*), where positions *a* and *d* are occupied by hydrophobic residues, often Val, Leu, or Ile. This pattern forms a hydrophobic core through “knobs-into-holes” interactions, which stabilizes the coiled-coil structure. The remaining positions (*b*, *c*, *e*, *f*, and *g*) are generally hydrophilic, contributing to the solubility and surface properties of the protein (5, 6).

Traditionally viewed as rigid structures, many coiled coils exhibit significant conformational flexibility (7, 8, 9, 10, 11). Approximately 20% of the *a* and *d* positions are occupied by polar or charged amino acids (12), which enables conformational plasticity. Eventually, this allows coiled coils to adopt different conformations, manifested as changes in the degree of supercoiling, rotational or translational changes, even transitions between parallel or antiparallel arrangements, or complete unfolding and refolding (6, 13).

As far as signal transduction is concerned, coiled coils transmit conformational changes in proteins, relaying signals from one domain to another, such as from sensor to effector domains (6). This is particularly relevant in proteins, such as adenylate cyclases (14), guanylate cyclases (15), and diguanylate cyclases (DGCs) (8, 11, 16, 17), where coiled-coil motifs enable activation of catalytic domains in response to external stimuli. DGCs featuring a GGDEF domain synthesize the secondary messenger cyclic di-GMP (c-di-GMP), a central signaling molecule in bacteria that regulates processes, such as biofilm formation, motility, and virulence (18, 19). For the GGDEF domain to be catalytically active, it must form a properly oriented homodimer, with the half active sites of each GGDEF protomer facing each other. This orientation is facilitated in the majority of GGDEFs by the linker helices and their architecture, which can be regulated by N-terminal sensory domains (18). Examples of coiled-coil–linked GGDEF domains include the phytochrome-regulated DGCs, such as *Is*PadC (PadC from *Idiomarina* species) (8, 16), and Rec-regulated DGCs, like DgcR (11). Two overlapping heptad repeat patterns can be identified in the linker region of these systems, each defining a distinct coiled-coil architecture. One pattern forms a stimulating coiled-coil register that activates the GGDEF domain, whereas the other forms an inhibiting coiled-coil register that suppresses enzymatic activity. The balance between these two registers determines the overall activity of the GGDEF domain, enabling the protein to dynamically regulate its function in response to environmental signals.

In addition to phytochrome or phospho-receiver sensory domains, LOV (light–oxygen–voltage) domains, a subset of the PAS (Per-ARNT-SIM) superfamily, can also be linked to GGDEF domains (20) and thereby allow blue light signals to regulate DGC activity. LOV domains, typically around 110 amino acids in length, use flavin chromophores as light-sensing molecules (21, 22). Upon blue light absorption, flavin undergoes a photochemical reaction, forming a covalent bond with a conserved Cys residue (23). This triggers conformational changes transmitted mostly through helical extensions at the N and C termini, often referred to as the A’α and Jα-helices, respectively, allowing LOV domains to regulate various effector proteins *via* modular domain arrangements (24, 25, 26, 27, 28, 29, 30), including not only GGDEF domains but also many others (20). Due to their modular nature and universally present cofactor, blue light–sensing LOV domains have been widely used as optogenetic input domains (20, 31, 32).

Our previous work explored naturally occurring LOV-activated DGCs (LadCs), leading to the characterization of the highly efficient *Methylotenera* sp. (*Ms*LadC) system, a prototypical LOV domain with a helical linker, and GGDEF effector domain (33). In the suggested model, light-induced changes destabilize a caged GGDEF architecture in *Ms*LadC, leading to the formation of an extended coiled-coil linker—previously disordered in the dark state—that optimally positions GGDEF domains for catalysis. This involves two key steps: release of the GGDEF domains bound in an unproductive conformation on opposite sides of the LOV dimer to allow coiled-coil assembly and formation of the stimulating coiled-coil register required for high DGC activity. Relative stabilities stimulating *versus* inhibiting coiled-coil register architectures by subtle variations in the linker sequence can be employed by nature to modulate the dynamic range as well as the specific activities (33). In this study, we aimed to identify key residues involved in this switch mechanism by focusing on residues at the inhibitory interface and special positions in the proposed coiled-coil linker helix. Using an *in vivo* DGC activity screen and *in vitro* characterization of interesting variants, we found that single or double amino acid substitutions at the inhibitory interface were generally not sufficient to relieve the caging mechanism, but that substitutions in the coiled-coil linker region were very effective in influencing activity. To further characterize the caging mechanism at the LOV–GGDEF interface, we generated chimeras by replacing the whole GGDEF domain as well as the LOV domain: both approaches resulted in constitutively active chimeras, which could, however, still be regulated by light *via* register switching of the *Ms*LadC linker region. Our findings not only highlight the central role of the helical linker in the *Ms*LadC switch but also newly demonstrate the involvement of the characteristic GGDEF inhibitory site (I-site) in the tight dark-state inhibited conformation. The combined results provide valuable insights for guiding and enhancing the performance in potential optogenetic applications of the molecular on–off switch *Ms*LadC.

## Results

### Establishing an *in vivo* diguanylate-cyclase activity assay

**Figure 1:**
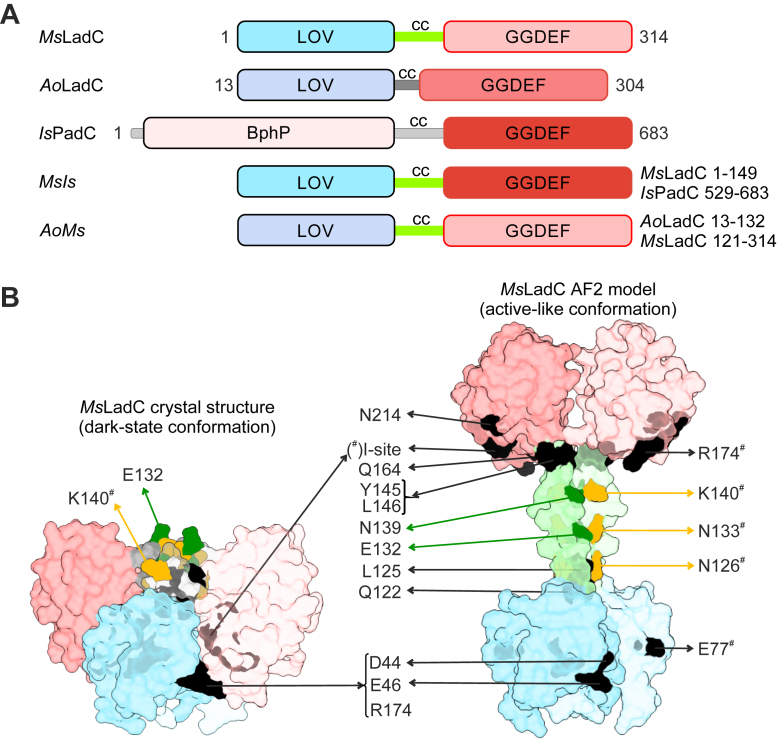
**Overview of studied *Ms*LadC variants.***A,* schematic representation of the domain architectures of naturally occurring *Ms*LadC, *Ao*LadC, and *Is*PadC as well as their chimeras *MsIs* and *AoMs*. LOV domains are colored in shades of *blue*, and GGDEF domains are colored in shades of *red*, depending on their origin. The three-domain bacteriophytochrome sensor of *Is*PadC is simplified as one BphP module in *dark green*. The colors help to appreciate the chimeras generated by swapping LOV and GGDEF domains between individual systems (*MsIs* and *AoMs* nomenclature follows the source of LOV–GGDEF domains, respectively). The coiled-coil linker region between sensor and effector domains is abbreviated with cc, and the *Ms*LadC cc is highlighted in *green*. *B,* the crystal structure of *Ms*LadC (*left*, Protein Data Bank code: 8C05) and the dimeric AlphaFold2 model (*right*) are shown as surface representations with individual domains colored according to *A*. Indicated targeted positions for substitutions are shown on the dimeric surface representations with one protomer surface made transparent, and its residues are denoted with a hashtag (^#^). Positions targeted to destabilize the inhibitory interface are colored *black*, whereas those targeted for register stabilization are colored *dark green* or *orange* for inhibiting and stimulating register stabilization, respectively. *Ao*LadC, LadC from *Aquella oligotrophica*; *Is*PadC, PadC from *Idiomarina* species; LOV, light–oxygen–voltage domain; *Ms*LadC, LadC from *Methylotenera* sp.

**Figure 2:**
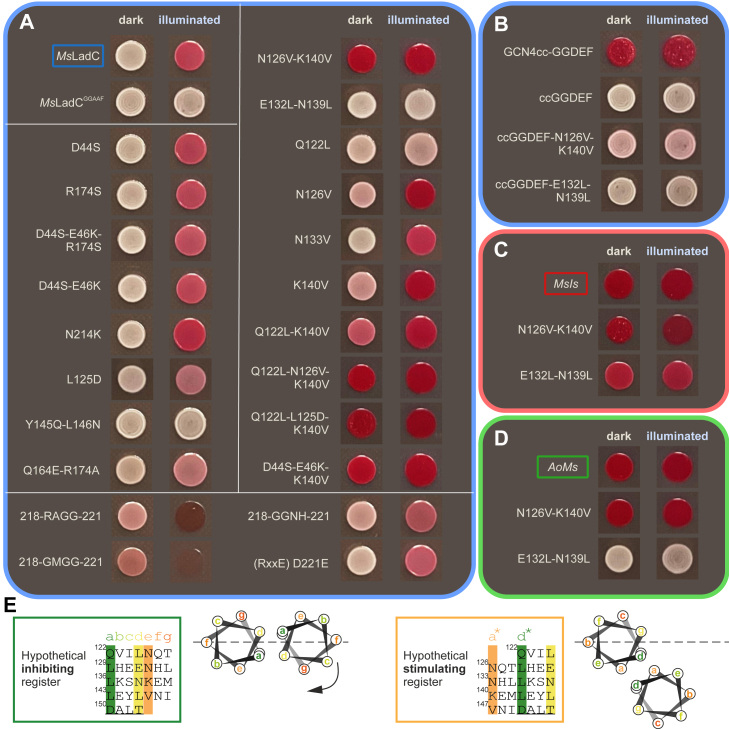
***In vivo* DGC activity assay.** The four blocks with different outline colors group variants according to the three systems of interest: (*A*) *Ms*LadC, (*B*) a subgroup of coiled-coil GGDEF truncations of *Ms*LadC, (*C*) *MsIs*, and (*D*) *AoMs* chimeras. We used *Ms*LadC as a positive control and a variant with compromised catalytic activity, *Ms*LadC^GGAAF^, as a negative control. *White lines* in *A* subdivide the variants according to positive and negative controls (*top left*), sensor–effector interface residues (*left column*), GGDEF inhibitory site variants (*bottom*), and coiled-coil linker substitutions (*right column*). In each case, the *left* colony was grown on a plate under dark conditions, and the *right* colony originates from a plate with constant *blue* light illumination. Uncropped plates are shown in Figure S2. *E,* shows the sensor–effector linker region of *Ms*LadC in two alternative coiled-coil registers represented as heptad repeats in the box and as helical wheel with an *arrow* indicating potential translational movement for register switching according to Gourinchas *et al.* (8). DGC, diguanylate cyclase; *Ms*LadC, LadC from *Methylotenera* sp.

We previously proposed a two-stage activation mechanism for *Ms*LadC (33). However, identifying key structural elements responsible for tight dark-state inhibition has proven to be challenging because of the extensive LOV–GGDEF inhibitory interface. Despite our previous attempts to alleviate dark-state inhibition through amino acid substitutions, such as *Ms*LadC-D44S-E46K and *Ms*LadC-R174S, none of these variants yielded sufficient amounts of purified protein required for *in vitro* experiments (33). To determine the effect of different amino acid substitutions on the functionality of the *Ms*LadC switch without the requirement of purifying all variants, we optimized an *in vivo* DGC activity assay based on previously established protocols (34, 35). In this assay, Congo Red binds to polysaccharides produced by *Escherichia coli* as a result of c-di-GMP production, causing the colonies to appear red (36). Nucleotide sequences corresponding to variants of interest and selected chimeras of LOV and GGDEF domains (Fig. 1*A*) were incorporated into the pET-M11 plasmid background, with an N-terminal His tag and a GB1 solubility tag–coding sequence. The GB1 tag proved essential to produce *Ms*LadC in *E. coli* (Fig. S1) and to visualize DGC activity under the conditions of the screening assay (Figs. 2 and S2). During the optimization of screening conditions (plate composition for optimal contrast, induction time with IPTG, incubation time, and temperature), reproducibility of the screening approach was confirmed multiple times, for example, by repeatedly observing light activation of the WT protein or clear results for positive and negative controls. Eventually, all constructs were spotted at identical cell densities, attempting to obtain comparable expression levels. With substantially differing protein sequences, especially for the chimeric proteins, variations in expression levels and the amount of soluble protein are likely to exist. Hence, the screening assay should only be considered as a qualitative tool for addressing overall catalytic activity and light regulation capacity.

We devised two groups of amino acid substitutions informed by structural data obtained from the dark-state crystal structure (33) and literature on coiled-coil switching mechanisms (8, 9, 11, 34, 35) (Fig. 1*B*). One group of substitutions targeted the interface between the LOV and GGDEF domains, crucial for the tight inhibition observed in the dark state. The other group focused on residues within the linker region, based on the hypothesis of two alternative coiled-coil registers. In this context, we introduced residues to stabilize either the stimulating register or the inhibiting register. In addition, we created an *Ms*LadC–*Is*PadC chimera (Fig. 1*A*, *MsIs*) by substituting the GGDEF domain of *Ms*LadC with that from the distantly related red light–regulated system *Is*PadC (16), aiming to disrupt the LOV–GGDEF-caging mechanism. Similarly, we also replaced the LOV domain of *Ms*LadC with that of an LadC homolog featuring a short coiled-coil linker length and likely no inhibitory interface based on our previous characterization (33), generating the *AoMs* chimera (Fig. 1*A*, *AoMs*).

### Addressing the dark-state inhibitory interface using the *in vivo* screen

Based on the dark-state crystal structure (Figs. 1*B* and S3*A*), we hypothesized that the formation of a salt bridge between Asp44 and Arg174 could be disrupted in the variant *Ms*LadC-D44S–E46K–R174S (Fig. S3*B*), potentially leading to increased dark-state activity. Similarly, the interaction between Asn214 and the LOV-domain backbone was expected to be affected by the insertion of a larger side chain, such as Lys, resulting in inhibitory interface disruption. In addition, Leu125 appears to engage in hydrophobic-stabilizing interactions at the inhibitory interface, which we attempted to disrupt by inserting a charged Asp residue (Fig. S3*C*). With *Ms*LadC-Y145Q–L146N or -Q164E–R174A, we also probed different combinations to alleviate the tight inhibition of the WT protein. However, most substitutions within the inhibitory interface group demonstrated behavior similar to the WT with slightly decreased or increased light-state activities (Q164E–R174A or N214K, respectively) or completely lost DGC activity, such as for the removal of the hydrophobic patch in the Y145Q–L146N variant (Fig. 2*A*).

We also attempted to disrupt the autoinhibitory site (I-site) of the GGDEF domain, characterized by an RxxD motif (18), 218-RGSD-221 in *Ms*LadC, which is buried in the inhibitory interface within a groove of the LOV dimer (Fig. S3*D*). Previous attempts to substitute Arg218 (37) resulted in reduced yield and overall protein fitness reduction, where viable variants did not show a promising shift of their conformations toward the active state. Since the I-site is dynamically coupled with the active site (34), we attempted to substitute the entire RxxD motif with various sequences that had previously shown relief of autoinhibition while maintaining DGC activity: GMGG, RxxE, RAGG, and GGNH (34). In search for destabilized dark-state inhibition, two of the *Ms*LadC variants, 218-GMGG-221 and 218-RAGG-221, indeed exhibited DGC activity under dark conditions in the *in vivo* assay (Fig. 2*A*). The other two variants, *Ms*LadC-D221E and -218-GGNH-221, retained more WT-like behavior *in vivo* while still featuring slightly increased dark-state coloration. In combination, this suggests that the GGDEF I-site contributes to the inhibitory mechanism more than previously anticipated.

### Applying the *in vivo* screen to linker register variants

To address the role of the coiled-coil linker for the *Ms*LadC GGDEF domain, we initially replaced the entire sensor and linker region with the GCN4 coiled coil, in analogy to a previously described artificial GCN4cc–GGDEF fusion (35). Similar to the findings of De *et al.*, we observed constitutively high DGC activity for this construct, whereas the isolated *Ms*LadC’s GGDEF domain construct, including the preceding natural linker sequence (*Ms*-ccGGDEF) showed no activity (Fig. 2*B*). In line with the heptad repeat pattern of GCN4 and register principles of *Is*PadC (16), we then substituted positions Gln122, Asn126, and Lys140 (position *a∗* in the stimulating register heptad repeat, Fig. 2*E*) in the full-length context to Leu or Val, aiming to stabilize the stimulating register. Single substitution variants *Ms*LadC-N126V and *Ms*LadC-K140V both increased basal dark-state and light-state *in vivo* DGC activities (Fig. 2*A*), whereas their combination yielded a constitutively highly active DGC (Fig. 2*A*). Substitution of Gln122, the most N-terminal part of the anticipated coiled-coil linker, with a hydrophobic amino acid led to almost no detectable DGC activity, even upon illumination (Fig. 2*A*). Interestingly, the variant *Ms*LadC-Q122L–K140V showed dark-state DGC activity and upregulated light-state activity (Fig. 2*A*), even in comparison to K140V alone. Such unexpected synergistic effects can be observed for some combinations, whereas others are more in line with additive effects, as for example, seen in the additionally increased basal activities of the Q122L–N126V–K140V triple variant (Fig. 2*A*). In addition to these substitutions, we also investigated the effect of the N133V variant on the *in vivo* DGC activity, since the corresponding position in GCN4 is also an Asn (35). *Ms*LadC-N133V, however, retained behavior similar to the WT (Fig. 2*A*), and the substitution of Asn133 apparently does not interfere with the register switching mechanism.

Furthermore, combining substitutions at the inhibitory interface with those in the stimulating register again led to the emergence of variants with unexpected synergistic effects indicated by high dark-state activities, such as in *Ms*LadC-D44S–E46K–K140V (Fig. 2*A*). Apparently, there is a coupling of conformational dynamics at the inhibitory interface and the coiled coil. Our observations here align with previously published *in vitro* characterization, where *Ms*LadC-Q122L showed significantly diminished DGC activities. The *in vivo* DGC activity of *Ms*LadC-K140V, however, shows a deviation from the *in vitro* data (33). Conversely, attempts to stabilize the inhibitory register by substituting Glu132 and Asn139 with hydrophobic residues (position *d* in the inhibiting register heptad repeats, Fig. 2*E*) resulted in no detectable DGC activity, that is, *Ms*LadC-E132L–N139L (Fig. 2*A*).

To further validate the observed effects of substitutions in the linker region, we also generated the isolated *Ms*LadC’s GGDEF domain construct, including the preceding linker (*Ms*-ccGGDEF) and introduced substitutions to stabilize either of the two possible heptad registers. The *Ms*-ccGGDEF construct neither did exhibit any DGC activity *in vivo* (Fig. 2*B*) nor did its variant with a stabilized inhibiting register (E132L–N139L). However, introducing substitutions that stabilize the stimulating register (N126V–K140V) resulted in moderate but constitutive DGC activity (Fig. 2*B*). As expected, none of the *Ms*-ccGGDEF variants displayed altered behavior upon illumination because of the absence of the photosensory domain. These outcomes demonstrated that the stimulating register configuration is crucial for DGC activity and that the preceding LOV domain additionally influences the coiled-coil architecture and/or its conformational dynamics.

### Insights obtained from individual sensor–effector chimeras

Replacing the entire GGDEF domain in the chimera, abbreviated as *MsIs* (*Ms*LadC-LOVcc–*Is*PadC-GGDEF), should prevent the formation of the characteristic LOV–GGDEF inhibitory interface, thereby enabling light regulation *via* register switching alone. Interestingly, we observed constitutively high *in vivo* DGC activity, making differences between dark and light conditions hard to assess (Fig. 2*C*). To probe the functionality of the register switching mechanism, we also introduced stabilizing substitutions targeting either of the two coiled-coil registers (Fig. 2*E*). The *MsIs*-N126V–K140V variant, designed to stabilize the stimulating register, did not show pronounced changes compared with the *MsIs* chimera (Fig. 2*C*), as the red color appears to be already saturated. In contrast, inhibiting-register stabilization led to a decrease in the *in vivo* DGC activity, observed by lighter red coloration and comparable levels for both the dark- and light-state *MsIs*-E132L–N139L (Fig. 2*C*).

We also characterized a chimera employing an LOV domain from a related LadC system with a shorter sensor–effector linker that is not able to form the inhibited dimer architecture based on previous results: *Aquella oligotrophica* LadC (*Ao*LadC) (33). As far as the screening results of *AoMs* (*Ao*LadC-LOV–*Ms*LadC-ccGGDEF) are concerned, the trend observed for *MsIs* could be mostly reproduced: high basal activity using the native *Ms*LadC linker sequence and continuously high activity in the *AoMs*-N126V–K140V variant. The *AoMs*-E132L–N139L variant, in contrast, showed full inhibition in both dark and light conditions (Fig. 2*D*). In combination, these results further highlight the importance of the inhibiting and stimulating coiled-coil registers, even in the absence of inhibitory interface contributions. Since additional effects that influence the observed activities, like GGDEF oligomerization, cannot be ruled out *in vivo*, we followed up with a biochemical characterization of interesting systems.

### Purification and biochemical characterization

Initial attempts to produce and purify variants, such as *Ms*LadC-D44S–E46K and *Ms*LadC-R174S, yielded inadequate protein yields for *in vitro* experiments because of increased protein aggregation and precipitation (33). Given the pivotal role of the coiled-coil linker region in the light-regulation mechanism, as demonstrated by the *in vivo* DGC assay, we directed our efforts to variants with stabilized coiled-coil register sequences and chimeras lacking the *Ms*LadC-specific dark-state GGDEF caging.

Utilizing a previously described purification protocol (33), we attempted to purify several constitutively active *Ms*LadC variants, including *Ms*LadC-Q122L–N126V–K140V as well as the *MsIs* and *AoMs* chimeras. Although we could confirm overproduction of several *Ms*LadC variants in bacterial cultures by SDS-PAGE analysis, we could not efficiently purify the corresponding proteins. All tested *Ms*LadC variants exhibited different tendencies to precipitate, aggregate, and/or retain impurities, making their biochemical characterization impossible. Due to these issues, we were only able to characterize *MsIs*, *AoMs*, and register variants of the latter in detail. To optimize the time requirements of the purification and to minimize protein loss, we characterized all systems without cleaving off the GB1-solubility tag, which helped to bypass some of the aggregation and precipitation issues. To allow a direct comparison with *Ms*LadC, we also characterized *Ms*LadC in the presence of the solubility tag, and all data presented in this article were obtained under these conditions.

Interestingly, while *MsIs* could be purified efficiently and showed a normal LOV photocycle (see later), the corresponding coiled-coil stabilizing variants (*MsIs-*E132L–N139L and *MsIs-*N126V–K140V (abbreviated as EN and NK, respectively, subsequently) obtained after nickel–nitrilotriacetic acid and desalting steps were colorless. Attempts to reintroduce flavin to these *MsIs* variants were unsuccessful, as the proteins did not demonstrate any noticeable affinity for flavin. These variants were also prone to oligomerization and eventually aggregation, preventing their more detailed characterization. Since the flavin binding pocket of the LOV domain is close to the inhibitory interface, we assumed that the absence of the caged conformation might influence the integrity of the flavin–LOV interaction—especially in variants stabilizing the elongated coiled-coil architecture in constructs encompassing the *Ms*LadC LOV domain. The *AoMs* chimera, featuring a LOV domain originating from a system without an inhibitory LOV–GGDEF interface, indeed behaved differently. In this system, the coiled-coil stabilizing variants could also be expressed, purified, and characterized in more detail (see later).

#### UV–visible spectroscopy

**Figure 3:**
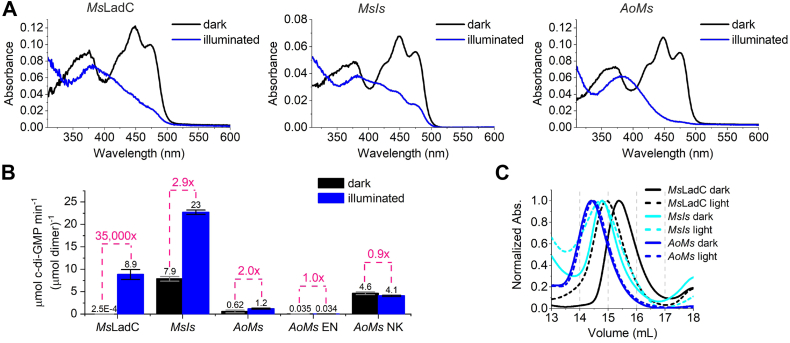
**Biochemical characterization of *Ms*LadC and LOV–GGDEF chimeras.***A,* UV–visible absorption spectra comparing dark and light states of *Ms*LadC, *MsIs*, and *AoMs. B, in vitro* DGC activities at 200 μM GTP under dark and illuminated conditions. Activities were normalized to the concentration of dimeric protein. Initial rates of product formation were calculated from linear fits of GTP conversion progress curves. Error bars represent standard errors of the linear regression estimates, based on sample standard deviations from triplicate measurements. *C,* size-exclusion chromatography profiles of dark and light states of *MsIs* and *AoMs* compared with *Ms*LadC. For better visualization, only normalized 280 nm traces are shown for the elution volume of interest between 13 and 18 ml. LOV, light–oxygen–voltage domain; *Ms*LadC, LadC from *Methylotenera* sp

We examined the spectral properties and the *in vitro* DGC activities of purified *Ms*LadC, *MsIs*, *AoMs* and their linker variants. All systems retained functional photocycles (Figs. 3*A*, S4, *A*–*H*), exhibiting similar thermal recoveries for *Ms*LadC and *MsIs*, with mean lifetimes of 95 ± 1 s and 121 ± 1 s, respectively. *AoMs* features a substantially slower recovery with a mean lifetime of 17.2 ± 0.1 min, more resembling the *Ao*LadC WT (33). Also, the linker variants of *AoMs* fall into the same recovery regime with 12.4 ± 0.4 min and 19.3 ± 0.1 min for *AoMs* EN and *AoMs* NK, respectively (Fig. S4).

#### *In vitro* DGC activity assays

Similar to observations from the spectroscopic characterization, the presence of the GB1 solubility tag also slightly affected the obtained kinetic parameters, resulting in ∼two-fold decreased *in vitro* DGC activity for *Ms*LadC compared with previously determined values (dark: 0.0007 ± 0.0002 μmol c-di-GMP min^−1^ [μmol *Ms*LadC_2_]^−1^; illuminated: 14.1 ± 0.9 μmol c-di-GMP min^−1^ [μmol *Ms*LadC_2_]^−1^, at 200 μM GTP (33)), but the tight dark-state inhibition mechanism remained unaffected (Fig. 3*B*). This was consistent with the *in vivo* activities, where a remarkably high fold change of activation is still observed.

*MsIs* showed higher overall DGC activity in the *in vitro* enzymatic activity assay, consistent with expectations based on the *in vivo* assay, particularly in the light state (Fig. 3*B*). Dark-state *MsIs* also demonstrated increased *in vitro* DGC activity compared with the *Ms*LadC, in line with the elimination of the inhibitory interface. However, this activity was comparable to that of the light-state *Ms*LadC, which was not observed in the *in vivo* DGC assay. It is important to note that comparing *in vitro* and *in vivo* activities requires caution, as *in vitro* reaction conditions are protein concentration normalized, whereas *in vivo*, intracellular levels and forms of functionally relevant species may vary for each variant. In addition, pH, salt concentrations, and the effect of crowding can vary strongly between cellular and *in vitro* environments. In line with this, dark-state *MsIs* displayed very strong red coloration *in vivo* but lower specific activity than light-activated *Ms*LadC *in vitro*. The overall fold change of light activation *in vitro* in *MsIs* was reduced to approximately threefold, and it demonstrated the typical product inhibition at higher substrate concentrations (Fig. S5), as expected for the GGDEF domain of *Is*PadC (38). For *MsIs*-E132L–N139L and -N126V–K140V, we detected the presence of c-di-GMP in the initially purified fraction, similar to previous observations for an *Is*PadC ccDGC construct (39), which had not been seen in *Ms*LadC variants before. Unfortunately, insufficient stability and thus low protein amounts precluded further characterization of these two variants.

**Figure 4:**
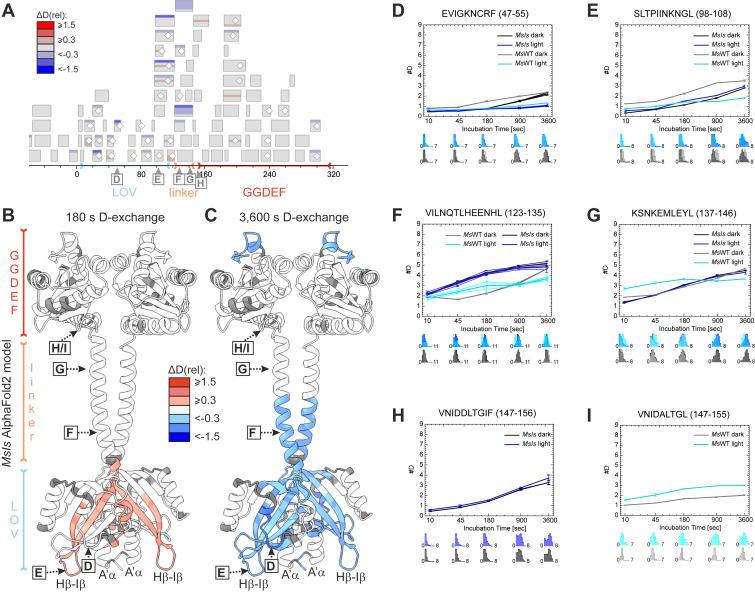
**Conformational dynamics of *MsIs*.***A,* peptide map illustrating differences in D uptake between dark and light state. The *x*-axis corresponds to residue numbers referenced to the LOV domain of *Ms*LadC. Negative values correspond to the preceding GB1-tag and linker sequence. Coloring corresponds to the differences in relative deuterium uptake of individual peptides according to the bar legend (54). *Diamond symbols* on individual peptides denote their respective MS/MS confirmation. *B* and *C,* color-coded *cartoon* of the *MsIs* AlphaFold2 model shows differences in D uptake (ΔD_rel_) after 180 s and 1 h of deuteration. *D*–*I, D* uptake curves of selected *MsIs* peptide fragments (*black* and *blue*) in comparison to the corresponding fragments of WT *Ms*LadC without GB1 tag (*gray* and *cyan*) measured previously (33). Mean values from three independent measurements are plotted, with error bars showing sample standard deviation. The lower plots display the distribution of deuterium levels from undeuterated to fully deuterated amide positions. LOV, light–oxygen–voltage domain; *Ms*LadC, LadC from Methylotenera sp.

For *AoMs*, the overall activities were lower than for the *MsIs* system; still, the *AoMs* chimera also showed no tight dark-state inhibition, suggesting a lack of the inhibitory interface. In fact, compared with *Ms*LadC, the specific dark-state activity is increased by a factor of 2500. Similarly to *MsIs*, a twofold increase in DGC activity was observed upon blue light illumination. Interestingly, the linker variants stabilized in the stimulating (*AoMs*-N126V–K140V) and inhibiting register (*AoMs*-E132L–N139L) showed either constitutively high or very low DGC activity, respectively. While their stability was good enough for the activity assays performed at low enzyme concentrations, a continued tendency to aggregate was still observed in the photometric analysis of dark-state recoveries (Fig. 4, *B* and *E*), especially for *AoMs*-EN. As aggregation appears to happen in almost all systems characterized in this context, the culprit for initial oligomerization could also be the linker region forming higher order coiled coils itself.

### In-solution structural changes

To confirm previously observed conformational changes of the *Ms*LadC switch (33) and to ensure consistency across experiments, we assessed the GB1-tagged version of *Ms*LadC, as all analyses in this study are based on tagged protein versions. We observed a subtle shift in the elution volume of dark-adapted dimeric *Ms*LadC because of the presence of the solubility tag. Specifically, this resulted in a lowered elution volume (15.4 ml) compared with the protein without the GB1 tag (33) under identical buffer and instrument settings. More importantly, the elution volume shifted further (14.9 ml, Figs. 3*C* and S6) upon blue light illumination, indicating a more extended conformation: in line with previous quantitative size-exclusion chromatography (SEC)–multiangle light scattering experiments of *Ms*LadC without GB1 tag that confirmed no changes in oligomeric state upon illumination (33). Hence, the previously characterized global conformational changes upon light activation remain unaffected by the presence of the solubility tag.

We then investigated the behavior of the *MsIs* and *AoMs* chimeras. Both dark and light states of *MsIs* and *AoMs* showed almost identical retention volumes (14.8/14.7 ml and 14.4/14.4 ml, respectively, Fig. 3*C*), with differences in absolute absorbance values at 280 nm because of the lower extinction coefficient of the flavin photoadduct (Fig. S6). While these retention volumes likely correspond to the illuminated active conformation of *Ms*LadC with an elongated architecture and hence the absence of a caged inhibitory conformation in both chimeras, the main emphasis should be put on the lack of light-induced structural rearrangements upon illumination for both chimeras. In addition, *MsIs* revealed its tendency to oligomerize and aggregate, as demonstrated by additional higher molecular weight peaks in the elution profile (Fig. S6*B*).

### Conformational dynamics (hydrogen–deuterium exchange mass spectrometry)

To further address molecular mechanisms governing the *Ms*LadC system, we also tried to crystallize the aforementioned constitutively active variants. The quickly recovering light state and the presumed multiple conformational states of the active conformation (as previously observed in small-angle X-ray scattering analysis of *Ms*LadC (33)), however, hindered our success in obtaining crystals. Moreover, tendencies of aggregation might cause significant challenges for small-angle X-ray scattering experiments. In light of these obstacles, we turned to hydrogen–deuterium exchange mass spectrometry (HDX–MS) as an alternative approach to identify structural elements involved in intramolecular signal transduction and help us understand how modifications to the amino acid sequence affect these functional regions. HDX–MS also offers insights into distinct conformational characteristics, such as the bimodal deuterium distribution observed in the linker region of *Ms*LadC (33).

Due to its higher dark-state activity and the availability of reference HDX–MS data for *Ms*LadC (33) and *Is*PadC (16), we focused on *MsIs* for these experiments. HDX–MS was employed to test whether the *MsIs* chimera, despite its limited fold change in enzymatic activation, reveals additional important regulatory elements by characteristic changes in conformational dynamics in addition to those identified in *Ms*LadC previously. Especially since it already adopts an elongated conformation in the dark, as demonstrated by the absence of significant light-induced global structural changes observed in the SEC analysis. This observation was further supported by the HDX–MS peptide maps illustrating differences in deuterium (D) uptake between dark- and light-state *MsIs* (Fig. 4*A*). Specifically, peptide maps show fewer colored fragments and more subtle changes for *MsIs*, indicating a lack of large-scale conformational rearrangements, compared with *Ms*LadC (33). In the following lines, we focus on *MsIs* observations generated under experimental settings similar to *Ms*LadC–*Is*PadC, differing only in salt concentration. Changes in conformational dynamics because of chimera generation go beyond those affected by subtle experimental differences but are not discussed in detail because of the combinatorial complexity of multiple comparisons that might distract from the scope of this article. We plotted D uptake differences between dark- and light-state *MsIs* onto the elongated AlphaFold2 model of *MsIs* (Fig. 4, *B* and *C*), generated in AlphaFold multimer mode and relaxed using the ROSIE Server (40). The relative D uptake levels (ΔD_rel_) upon blue light illumination are represented by the color spectrum ranging from red (indicating increased D uptake) to blue (indicating decreased D uptake). Upon illumination, we observed a subtle, yet distinct, increase in conformational dynamics of the LOV domain at the initial time points (ΔD_rel_ ca. 0.3, Fig. 4, *B* and *D*,E), particularly in regions such as the Cα–Dα loop and Hβ and Iβ strands. Over longer incubation periods, however, lower D uptake upon illumination was evident in regions, such as the Dα–Eα loop and the β-sheet core of the LOV domain (Fig. 4*C*). As seen in Figure 4*D*, light clearly activates the LOV domain in *MsIs*, yet the different output domain and linker architecture affect the conformational dynamics surrounding the adduct forming cysteine. The first half of the linker region showed similarly high conformational dynamics in both dark and light states of *MsIs* (Fig. 4*F*). Interestingly, the second half of the linker region showed dynamics very similar to the dark-state WT (Fig. 4*G*). The exchange kinetics in the DxLT wide turn at the N terminus of the GGDEF domain were again similar between the dark and light states of *MsIs* (Fig. 4*H*), unlike the previously observed significant differences seen in *Ms*LadC (Fig. 4*I*). The GGDEF domain exhibited only a few discernible differences, with minor effects in regions such as the α^1^–α^2^ loop and stabilization of the C terminus (α^4^). In summary, the most notable differences were concentrated in the LOV core and the N terminus of the linker region, highlighting their roles in the signal transduction of the *Ms*LadC switch and, to a lesser extent, in *MsIs*. These results confirm minimal light–induced changes, supporting the loss of inhibitory interface contacts and validating the mechanistic model.

## Discussion

Multidomain proteins employ sophisticated mechanisms for allosteric signal integration and regulation of specific functions. For this purpose, specific structural elements, such as coiled-coil structures, play a crucial role in regulating the assembly and function of multidomain proteins. Coiled coils facilitate “communication” between sensor and effector domains, as for example, the LOV and GGDEF domains in this specific study. The DGC activity of the GGDEF domain and the importance of c-di-GMP signaling networks in bacteria enabled us to screen for the effects of amino acid substitutions on *Ms*LadC functionality directly in *E. coli*. Building on our previously proposed two-stage activation mechanism for *Ms*LadC (33), we designed and analyzed several variants targeting the inhibitory interface and/or the linker heptad repeats. Our results confirm that the linker helices are key modulators of *Ms*LadC activity and suggest that the GGDEF’s I-site contributes to tight inhibition *via* the characteristic inhibitory interface observed in the *Ms*LadC structure (33).

### The stimulating register of the coiled coil is a requirement for DGC activity

We previously postulated that the active state of *Ms*LadC features a coiled-coil linker in a specific heptad register (33). Here, we confirmed that single amino-acid substitutions that stabilize the linker in a stimulating coiled-coil register significantly enhance DGC activity. The *Ms*LadC-N126V and *Ms*LadC-K140V variants exhibited increased enzyme activity upon illumination compared with the WT, whereas their combination resulted in constitutive DGC activity, reflecting their synergistic effect. We observed the same activating effects in *Ms*-ccGGDEF-N126V–K140V as well as in *MsIs*- and *AoMs*-N126V–K140V. Conversely, stabilizing the inhibiting register through E132L and N139L substitutions resulted in the complete absence of DGC activity in E132L–N139L variants of *Ms*LadC, *Ms*-ccGGDEF, and *AoMs*. Furthermore, E132L–N139L substitution reduced *in vivo* activity in constitutively active *MsIs*. These results demonstrate that simply releasing the GGDEF domains from the inhibitory interface alone is not sufficient for full GGDEF activation and support the notion that the coiled-coil register switch controls DGC activity in addition (8, 11). This is also reflected by the *Ms*-ccGGDEF construct, which alone did not exhibit any DGC activity *in vivo* unless the stimulating register was stabilized. Apparently, dimeric LOV domains support the transition to the active, stimulating coiled-coil conformation and/or stabilize its conformational dynamics.

While the term “binary switch” has been used to describe the register-switching mechanism in *Is*PadC (8) and DgcR (11), the regulatory mechanism in *Ms*LadC switch is more complex and involves an additional level of regulation. Nature apparently evolved the linker region of *Ms*LadC to populate an additional distinct conformation: a partially disordered linker stabilized by extensive direct interactions between sensor and effector domains.

In the context of the linker region, one noteworthy variant is *Ms*LadC-Q122L, which showed no *in vivo* DGC activity. Our previous characterization of *Ms*LadC-Q122L, although after tobacco etch virus cleavage, also showed significantly reduced DGC activities (33). Given that Gln122 is positioned at an *a* or *d∗* in the inhibiting or stimulating register, respectively, substitution to Leu was not expected to strongly shift the equilibrium to either dark state or light state. However, the observed effect suggests that the substitution Q122L may influence additional regions, potentially affecting the proper alignment of LOV protomers within the dimer (33). Without additional structural information, however, definitive conclusions are not feasible. Interestingly, this position is conserved as Arg in many LadC homologs (33) that could form a salt bridge with the conserved Glu96. A similar but inverted salt bridge has also been observed in *Ba*LOV-HK (41). In combination with K140V or N126V–K140V, Q122L also showed a stronger influence and synergistically contributed to stabilization of the stimulating register and increased DGC activities.

### Potential role of I-site in the inhibitory interface

Contrary to our expectations, destabilization of the inhibitory interface by selected amino acid substitutions did not significantly shift the equilibrium toward the active state. Most variants behaved similarly to the WT unless we eliminated all potential contacts by replacing the entire GGDEF domain, as reflected in the *MsIs* or *AoMs* chimeras. *MsIs*, with the GGDEF domain from the *Is*PadC system (16), and AoMs, with the *Ao*LadC LOV domain, presumably not forming inhibitory interface contacts, both demonstrated the interchangeable nature of modular domains. Proper positioning *via Ms*LadC’s linker helices allowed LOV and GGDEF domains to be swapped, yet to remain active and even regulated.

Although we could not pinpoint specific contacts of the I-site within the inhibited crystal structure, growing evidence suggests that the I-site also participates in protein–protein interactions (42, 43). Therefore, we modified the I-site in *Ms*LadC. Previous attempts to target Arg218 residue of the I-site (RxxD motif) were detrimental to purification success and DGC activity (37). Thus, we tested a set of four I-site variants previously shown to affect product inhibition without being detrimental to DGC activity (34). Two of these four variants, GMGG and RAGG, indeed shifted the equilibrium toward more active conformations in the dark state. Both variants feature Gly residues in the second part of the I-site. We hypothesize that the presence of Gly residues reduces steric constraints, destabilizing the interaction within the groove between the two LOV domains, which is needed for tight inhibition. This suggests that the I-site architecture, rather than a specific residue, is crucial for proper inhibitory interface formation under nonactinic conditions. While other contacts likely contribute to the overall stability of the inhibitory interface as well, they do not appear to be as significant as the I-site. Upon light activation, the increased red coloration in the *in vivo* assay for these I-site variants may result from the loss of autoinhibitory function, although this was not explicitly evaluated. The complex interplay between the I-site’s participation at the inhibitory interface, product feedback inhibition, and its allosteric coupling to the active site (34), as well as involvement in potential additional protein–protein interactions (42, 43), makes it difficult to accurately determine its suggested function in the *Ms*LadC switch mechanism. Yet, this elaborate system with several layers of allosteric regulation ensures the extremely high fold changes of *Ms*LadC activation.

### Insights from the *MsIs* and *AoMs* chimeras

The elimination of inhibitory interface contacts in *MsIs* and *AoMs* resulted in constitutively active DGCs, observed in both *in vivo* and *in vitro* assays. SEC analysis confirmed that both chimeras are likely extended dimers as they eluted similarly to the proposed extended form of *Ms*LadC (33). Combining these observations with *in vitro* enzymatic activities, we speculate that the residual twofold to threefold light regulation in *AoMs* and *MsIs*, respectively, is likely because of coiled-coil register switching. The minor fold changes might also explain why no significant difference between light and dark conditions was visible *in vivo*. Discrepancies observed between *in vivo* and *in vitro* DGC activities likely arise from variations in protein expression levels, aggregation, and assay conditions.

Analysis of conformational dynamics using HDX–MS revealed that light-induced changes in *MsIs* were primarily concentrated in the LOV core region and the linker. While the LOV domain of *MsIs* showed a decrease in conformational dynamics similar to *Ms*LadC, the extent of these changes was less pronounced. A subtle increase in conformational dynamics was observed upon illumination in regions such as the Cα–Dα loop, Hβ and Iβ strands, likely because of rearrangements resulting from the Gln flip (26). The linker helices in *MsIs* exhibited high conformational dynamics but showed no significant difference between dark and light states, reflecting a lack of structural rearrangements upon illumination. This suggests that the compact inhibited conformation is not formed and that the coiled coil is rather dynamic. Although some linker peptide fragments did show minor stabilizing effects upon illumination at longer incubation times, which could be indicative of changes in the linker register, these changes were less pronounced than in the *Is*PadC linker region (16). The *Ms*LadC linker may be optimized for conformational switching between a partially disordered state, maintaining the inhibitory interface, and an extended coiled-coil configuration rather than discrete register switching. Nonetheless, a stimulating register is essential for optimal DGC activity, as discussed above. Unlike the bimodal deuterium distributions observed in the linker region of the light-state *Ms*LadC (33), all detected fragments of *MsIs* exhibited typical unimodal deuterium distributions, indicating the absence of multiple conformations associated with the formation and release of the inhibitory interface and the corresponding transitions between a partially unstructured and fully extended coiled-coil linker region, respectively. In the GGDEF domain, regions involved in the inhibitory interface in *Ms*LadC, such as the wide-turn DxLT motif, previously showed strong changes in exchange rates (33). However, comparable changes did not occur in *MsIs*, also indicating the loss of inhibitory interface interactions. Functionally important regions of the GGDEF domain, such as substrate-binding elements, also showed no prominent changes in *MsIs*, which reflects its constitutive activity. This behavior in GGDEF regions contrasts with the observations in *Ms*LadC, where substantial structural rearrangements occurred, and, to a lesser extent, also in *Is*PadC (16).

### Possible functional role of oligomerization propensity

While we successfully characterized several *Ms*LadC-based systems (*Ms*LadC, *MsIs*, *AoMs*, and their linker variants) *in vitro*, the characterization of some variants turned out very challenging. Especially addressing extended coiled-coil linker conformations highlighted the oligomerization and/or aggregation propensity of these systems. These observations might indicate an evolutionary fine-tuned balance between the inhibitory interface conformation and extended conformations. Variants designed to stabilize either the stimulating or inhibiting register exhibited low solubility and were predominantly retained in the insoluble fraction, forming inclusion bodies. It remains unclear whether this aggregation was caused by an increased propensity for interdimer contacts of the LOV–GGDEF interface, favored oligomerization of the coiled-coil segments (6), or because of c-di-GMP crosslinking (18). For example, the two *MsIs* register variants copurified with c-di-GMP, suggesting that crosslinking *via* product inhibition at their I-sites (18) may have contributed to their aggregation. A shift to higher oligomers upon illumination was also observed in natural systems like *Serratia fonticola* LadC (33), which shares the same linker length and high fold change of activation as *Ms*LadC. Importantly, protein concentration and light exposure influence the reversibility of oligomerization, with increasing oligomer sizes, while still soluble, decreasing the chances of reverting to the dimeric assembly upon recovering to the dark state. Depending on the system, there might be a fine line between soluble aggregate formation and precipitation. Reversible oligomerization is also important in some other cases, for example, the WspR system, where different oligomeric states regulate GGDEF domain activity (35). Oligomerization might thus act as an additional regulatory mechanism in long-linker LadC systems. Oligomerization could either enable or prevent DGC activity (35) or facilitate further interactions in the active or c-di-GMP-inhibited states (43, 44, 45).

### “Slippery” heptad repeats in protein regulation

Despite the recurrent nature of structural elements involved in signal integration and transduction across various systems containing LOV (24, 27, 41, 46, 47, 48) or GGDEF (8, 11, 35, 45, 49) domains, the prevailing allosteric mechanism—whether register switching, unfolding transitions, inhibitory interface formation, or oligomerization—is apparently context dependent. Factors such as linker length and composition, the specific effector domains involved, and the evolutionary adaptations unique to each organism influence which regulatory mechanism is dominating.

*Ms*LadC, *Is*PadC (8), DgcR (11), and other examples, like BvgS (50), exhibit “slippery” heptad repeats in their linker regions that allow for register switching. These repeats enable subtle structural transitions in response to stimuli, regulating protein functionality. While signaling helices have been previously described as a distinct group of coiled coils (2), “slippery” coiled coils capable of register switching do not necessarily adhere to the conservation motifs identified for signaling helices. However, they share a common mechanism of regulating protein function through structural transitions. The potential for identifying these “slippery” coiled coils using bioinformatic methods and classifying them as a distinct subgroup within coiled coils, separate from signaling helices (2), remains to be explored. Evidence (8, 11, 16, 20, 50), including findings from this study, suggests that register shifting is a widespread and common regulation mechanism in modular proteins. It provides an outstanding potential for evolutionary fine-tuning of activation fold changes as well as specific activities without the need to address the sensor or effector domains but solely by modulating the relative coiled-coil stabilities of the integrator module.

**Figure 5:**
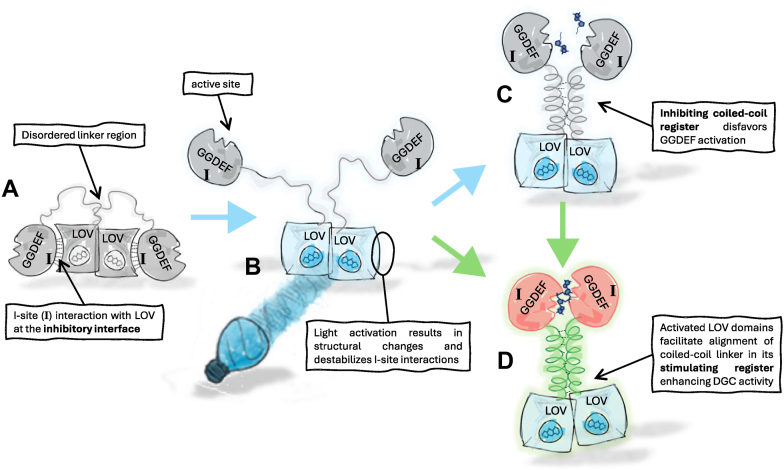
**Proposed mechanism of *Ms*LadC activation.***A, Ms*LadC preferentially adopts its inhibited conformation in the dark. Several interactions between the GGDEF I-site and LOV residues near the flavin binding pocket stabilize the caged GGDEF conformation in the dimeric context, preventing the encounter of active half-sites. The sensor–effector linker is partially disordered, and no continuous coiled-coil can be formed. *B,* upon illumination, structural and conformational changes in the LOV domain destabilize the caged assembly and allow the linker region to sample different coiled-coil architectures. *C* and *D,* depending on the coiled-coil sequence, the relative stabilities of inhibiting (*C*) *versus* stimulating (*D*) registers define the observed DGC activity and ultimately the fold change of the system. Only the stimulating register architecture enables optimal alignment of two GTP substrates in the active site formed by two GGDEF protomers. DGC, diguanylate cyclase; LOV, light–oxygen–voltage domain; *Ms*LadC, LadC from *Methylotenera* sp.

Altogether, our analysis indicated that activation of *Ms*LadC relies on appropriate stimulating register arrangement of the linker coiled coil, with high fold change and tight inhibition achieved through the dark state inhibitory interface (Fig. 5). The molecular understanding of the mechanistic details of such a two-stage activation mechanism enables future directed optimization of the *Ms*LadC system by tuning for specific dynamic ranges and/or fold changes. Considering the concept of conformational coupling between the I-site and the coiled-coil linker will be key to such endeavors. While challenges in the *in vitro* characterization highlight potential limitations of the *Ms*LadC system, insights gained from *in vivo* assays show that *Ms*LadC retains a tightly regulated switch mechanism in cells, pointing to its potential for optogenetic applications.

## Experimental procedures

### Sequence generation and site-directed mutagenesis

*MsIs* and *AoMs* chimeras were generated by cloning PCR-amplified fragments generated from previously described pET GB1 *Ms*LadC (33), pET *Is*PadC (16) and pET GB1 *Ao*LadC (33) plasmids (primers used are listed in Table S1) by Gibson assembly (51) using NEBuilder HiFi DNA Assembly. Substitution variants were produced by one-step site-directed mutagenesis (52) using pET GB1 *Ms*LadC, *MsIs*, or *AoMs*, or already generated variant plasmids as template (primers used are listed in Table S1).

### *In vivo* DGC assay

DGC activity was screened by adaptation of previously described protocols (34, 35). *E. coli* BL21 (DE3) cells transformed with pET GB1–based variants’ expression plasmids were grown at 37 °C in LB media with high salt supplemented with kanamycin (34 μg ml^−1^) to an absorbance (600 nm) of 0.5. Each culture was then induced with 0.1 mM IPTG for 2 h at 16 °C. Afterward, 2 μl of the induced culture (absorbance at 600 nm adjusted to 10) was spotted on LB-agar plates containing kanamycin (34 μg ml^−1^), Congo Red (0.05 mg ml^−1^), and Brilliant Blue G250 (0.02 mg ml^−1^), and incubated at 20 °C for 17 h in the dark or under constant blue light illumination (∼5 μW cm^−2^ at 455 nm). Then incubation proceeded at 4 °C for 3 days in the dark or under constant blue light illumination, before the pictures were taken. As a negative control, we used a pET GB1 *Ms*LadC^GGAAF^ construct.

### Protein production and purification

Plasmids containing the coding sequences of the different variants with additional N-terminal tobacco etch virus–cleavable 6His- and GB1-tags were transformed into RbCl-competent *E. coli* BL21 (DE3) cells and cultivated and harvested following the protocol described (33). Protein purification followed the same protocol described previously (33) with minor differences: N-terminal 6His-GB1-tags were not removed, and concentrated samples were desalted using PD-10 columns. Purified proteins were flash-frozen in liquid nitrogen and stored at −80 °C until needed. An additional purification step by SEC on a Superdex 200 Increase 10/300 GL column (GE Healthcare) equilibrated in the respective storage buffer (Table S2) was performed directly on the day of biochemical characterization experiments.

### Spectroscopic and enzyme kinetics characterization

UV–visible absorption spectra and recovery kinetics were measured as described (33), in the corresponding buffer (Table S2), at 20 °C. Briefly, blue light illumination was performed for 1 min with 1.2 mW cm^−2^ (465 nm Luminea LED). Recovery kinetics were followed at 20 °C by following the absorbance change at 448 nm, directly terminating illumination. No biological or technical replicates were performed in this case.

The conversion of GTP to c-di-GMP or pppGpG was measured by HPLC, as described (33). *MsIs* activities were determined at 1 μM protein for the dark state and 0.5 μM protein for the light state. *AoMs* activities were quantified from data measured with 2 μM protein in dark and light states. All time points were additionally quenched with 10 mM EDTA before denaturation. Each time point was prepared in triplicate. Absolute c-di-GMP amounts were quantified by the relative amount of product being formed with respect to the known GTP concentration. Sample standard deviations were then calculated for each point and contributed to the error estimation of the linear fit that was performed using Origin (OriginLab Corporation) 2019. All enzymatic activities were normalized to the concentration of the dimeric LadC.

### Hydrogen–deuterium exchange mass spectrometry

Deuterium exchange reactions were performed as described (33), and more details of the setup are provided (53). The *MsIs* stock solution (22 μM) was aliquoted (7 μl) in reaction tubes and equilibrated under nonactinic light (dim red or orange light) conditions at 20 °C for 1 min. For analyzing the illuminated state, samples were preirradiated with blue light (mounted LED, 455 nm, Thorlabs, 1.5 mW cm^−2^) for 1 min at 20 °C. Deuterium exchange reactions were started with a 1:15 dilution in deuterated buffer (10 mM Hepes [pD 7.0], 50 mM NaCl, and 2 mM MgCl_2_). Aliquots (15 μl) were removed and quenched at 10 s, 45 s, 3 min, 15 min, and 60 min with 15-μl ice-cold 200 mM ammonium formic acid (pH 2.5), 1.5 M urea, and flash-frozen in liquid nitrogen. Before injection into the HPLC system, aliquots were thawed by resuspension in ice-cold quenching buffer in a total volume of 50 μl. Samples of 80 μl were then injected into a cooled HPLC setup. Each time point was prepared in biological triplicates. Deuterium incorporation was analyzed and quantified with the Hexicon2 software (https://hx2.mr.mpg.de/) package (54). Sample standard deviations were calculated using Hexicon2 for the centroids of each peptide’s isotope pattern. Data are summarized in Table S3.

## Data availability

All data needed to evaluate the conclusions of the article are present in the article and/or the supporting information file. All data used in the analyses will be made available in the public repository of Graz University of Technology under https://doi.org/10.3217/a2dyr-0sa89.

## Supporting information

This article contains supporting information (33).

## Conflict of interest

The authors declare that they have no conflicts of interest with the contents of this article.
