# Supplementary material for: Coiled-coil register transitions and coupling with the effector’s inhibitory site enables high fold changes in blue light–regulated diguanylate cyclases
Source: J Biol Chem. 2025 Dec 6;302(1):111020. doi: 10.1016/j.jbc.2025.111020 (PMC12805367; doi:10.1016/j.jbc.2025.111020)
Supplement: Supporting information [file mmc1.pdf]

Coiled-coil linker register transitions and coupling with the effector's inhibitory-site enables high fold changes in blue light-regulated diguanylate cyclases

Uršula Vide[1], Gabriela Shickle[1], Julia Schwekendiek[1], and Andreas Winkler[1,2]

Affiliations
[1] Graz University of Technology, Graz, Austria
[2] BioTechMed Graz, Graz, Austria

Supporting information

Figures S1 – S6

Tables S1 – S3

Running title

Conformational coupling in sensor-diguanylate cyclases

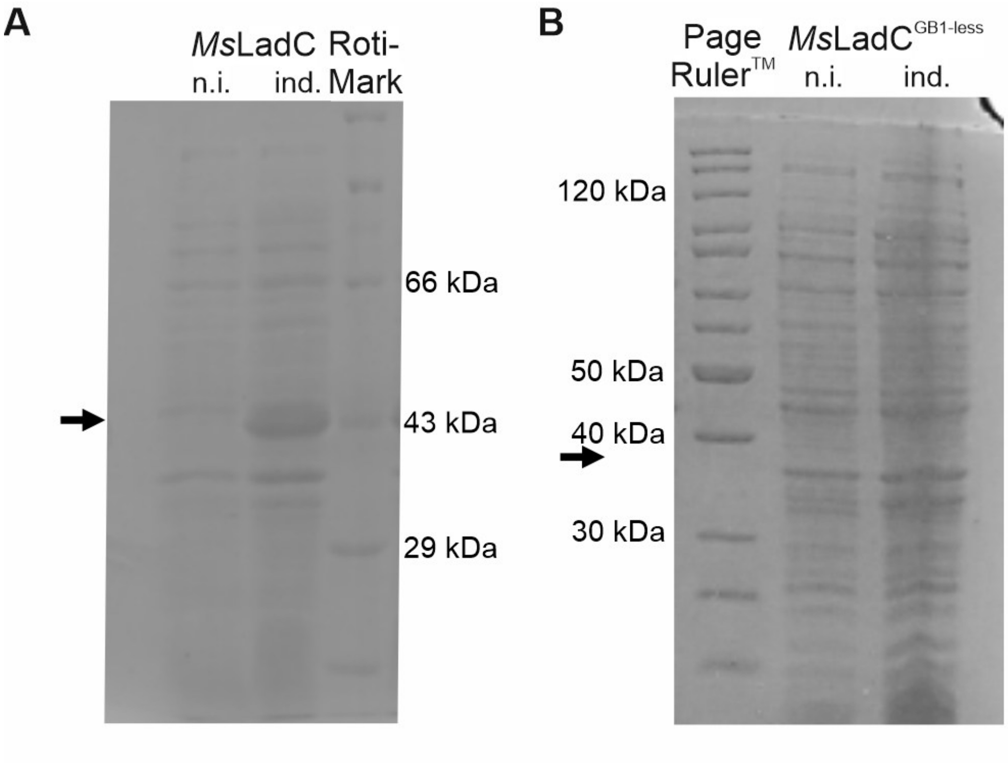

Figure S1: SDS-PAGE analysis of GB1-tagged MsLadC vs only His-tagged MsLadC*. Panel A shows three lanes; from left to right: uninduced control (n. i.), IPTG-induced cells (middle) and a protein marker (right). Panel B shows a similar layout with a different protein marker in the left lane of a different SDS-PAGE gel. Arrows indicate the expected molecular masses of GB1-MsLadC in panel A and His-MsLadC in panel B.*

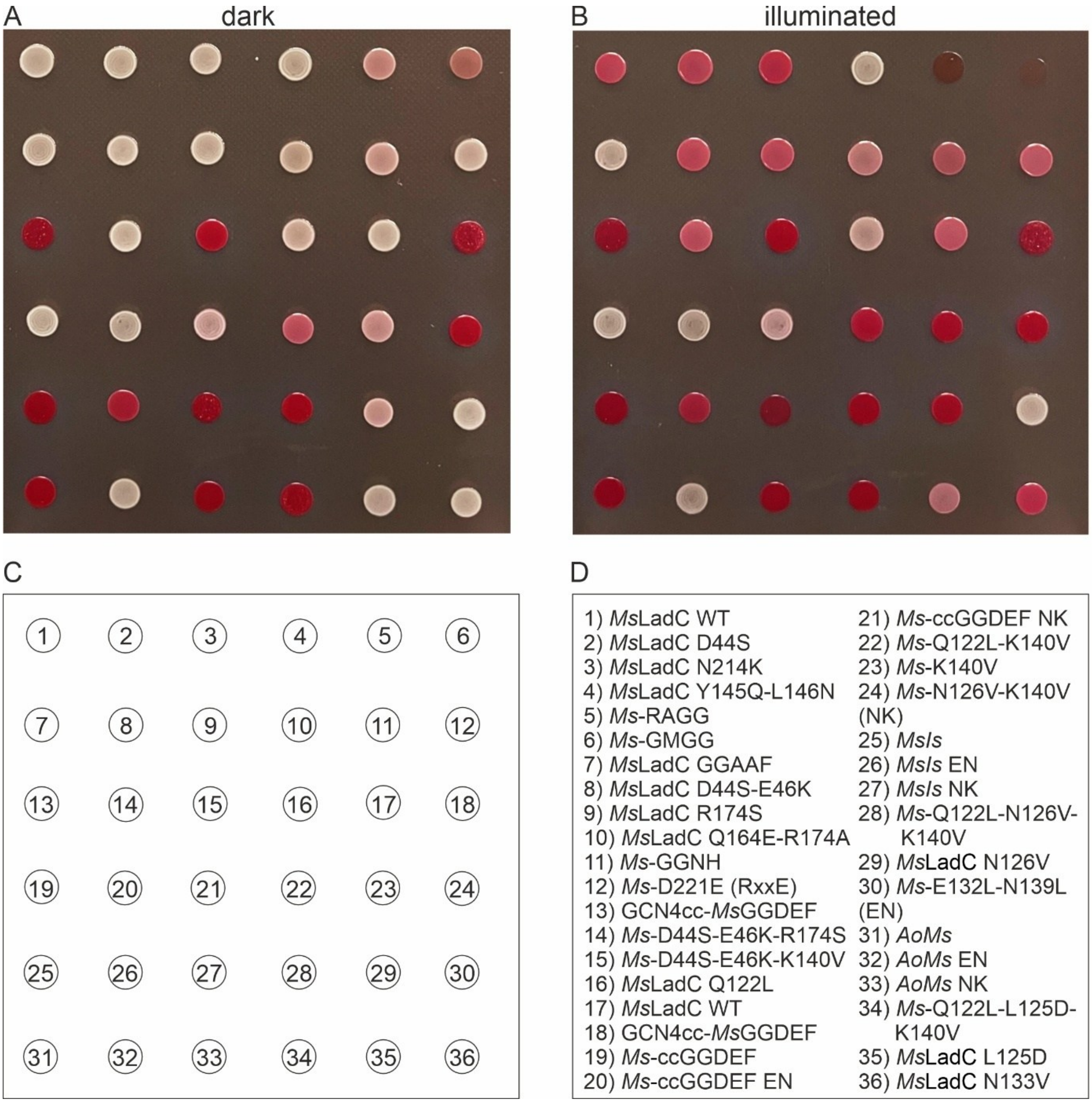

***Figure S2: In vivo DGC activity assay**. Uncropped images corresponding to the data shown in **Fig. 2A-D**. Panel **A** shows the plate incubated in the dark and panel **B** the corresponding illuminated plate. Panels **C** and **D** provide the legend to link individual spots to specific protein constructs. Off-white colors correspond to no/low c-di-GMP production and red colors to increased diguanylate cyclase activities.*

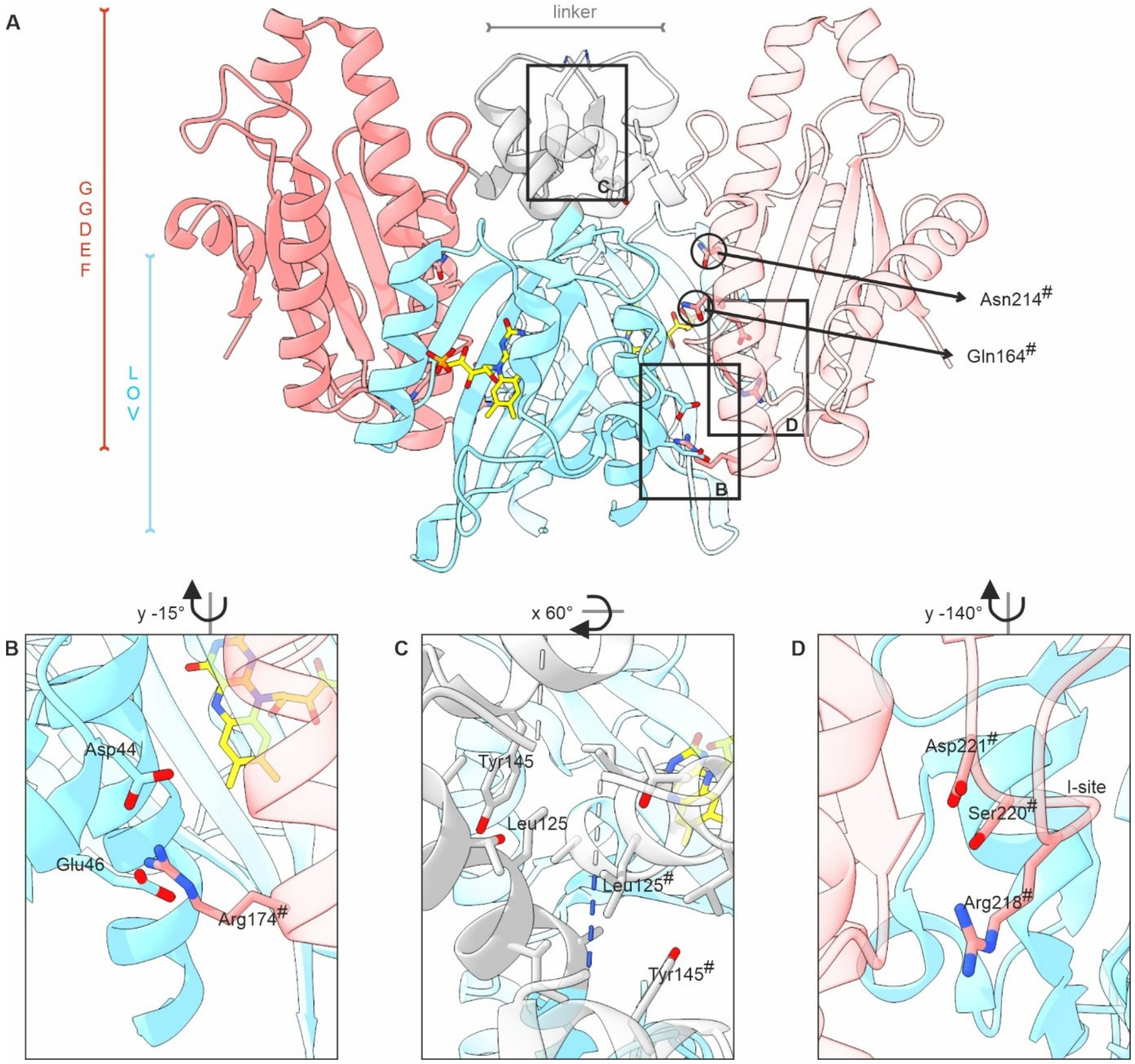

***Figure S3: Inhibited dark-state MsLadC structure-informed rational design for destabilization of the inhibitory interface**. **A**) The dark state is characterized by caging of GGDEF domains (in red) in the dimeric assembly to prevent their productive encounter for catalysis. Side chains targeted for substitution, and FMN, are shown as sticks. One protomer is shown in transparency and its residues are highlighted with a hashtag ($^{\#}$); **B-D**) rotated close up views for three interactions targeted by site-directed mutagenesis: **B**) the Asp44-Arg174$^{\#}$ region, **C**) the linker region with Leu125 and Tyr145, **D**) the GGDEF I-site RxxD (residues 218-221).*

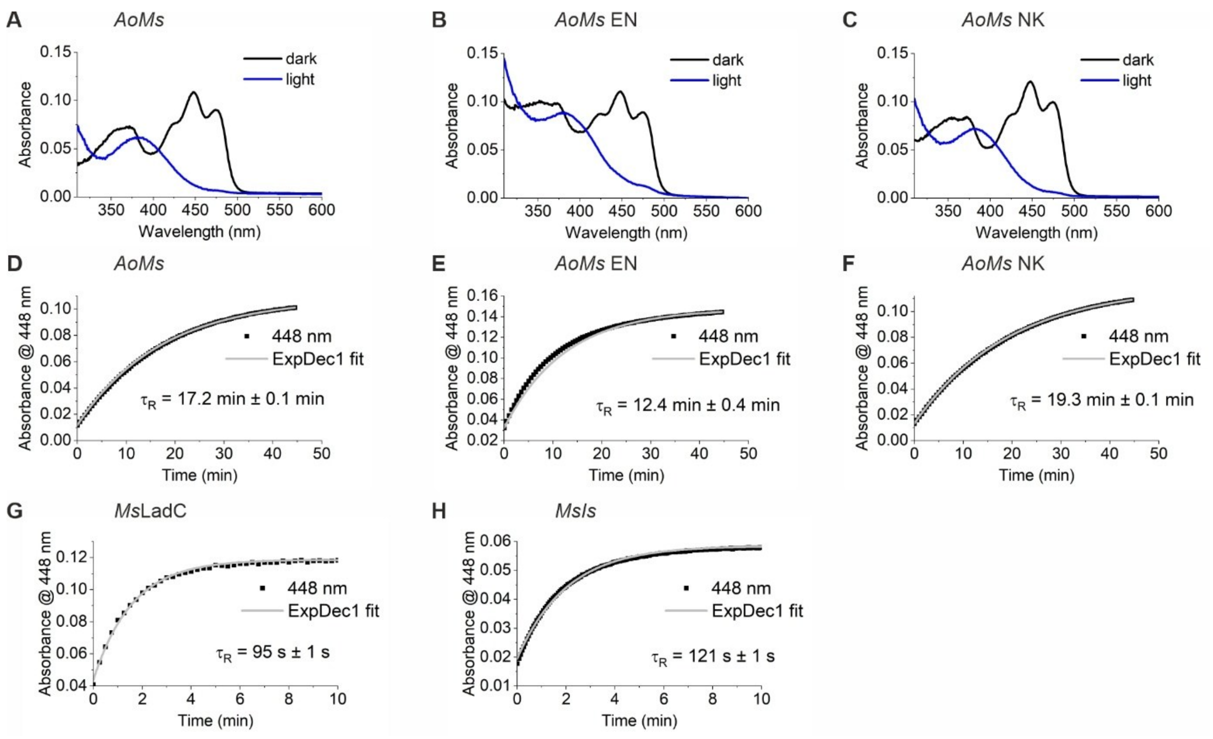

Figure S4: Biochemical characterization of MsLadC and associated chimeras*. *A-C*) *UV-Vis absorption spectra comparing dark and light state of AoMs and its EN and NK variants (E132L-N139L and N126V-K140V, respectively);* ***D-H*) Dark-state recovery kinetics were fitted with a single exponential function, shown in grey. Mean lifetimes (τ_R) and standard errors of the fit for single measurements are indicated in each panel.*

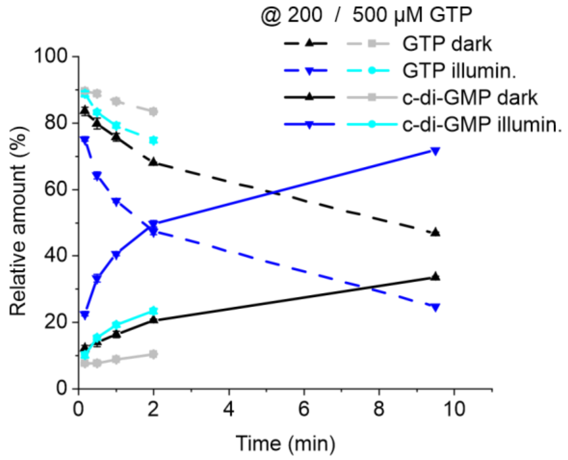

***Figure S5: Product inhibition in the MsIs chimera**. Shown are GTP conversion-progress curves at 200 and 500 µM GTP measured with 2 µM MsIs in dark conditions or under blue light.*

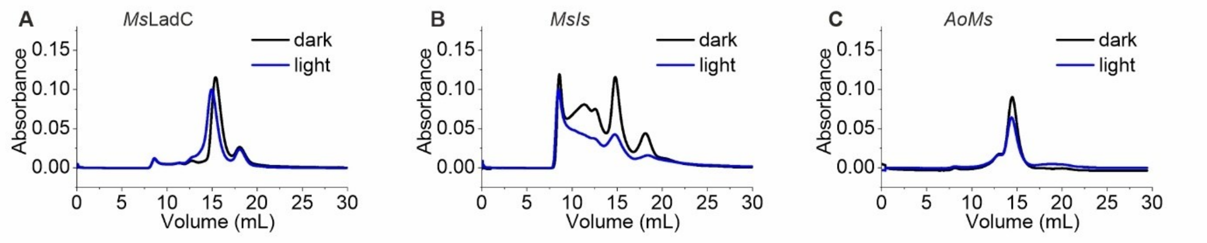

Figure S6: In-solution behavior of MsLadC variants. *SEC chromatograms of MsLadC (**A**), MsIs (**B**) and AoMs (**C**). Shown are 280 nm traces for samples kept in the dark in comparison to pre-illuminated samples with constant blue light illumination during the run.*

Table S1: Primers used for generation of the MsIs and AoMs chimeras as well as site-directed mutagenesis of MsLadC, MsIs, and AoMs*. Primers were also combined to create various multiple-substitution variants. Additionally, primers from (33) were used.*

	**Forward 5'->3'**	**Reverse 5'->3'**
pET GB1a *Ms*LadC-LOVcc	TAAGCGGCCGCACT	AATATTCACCAGATATTCCAGCATC
*Is*PadC-GGDEF	CAAAGAGATGCTGGAATATCTGGTGAATATTGATGATCTGACCGGTATCTTTAATCG	GAGTGCGGCCGCTTACTGGCTACAAACCTGATTACGAC
*Ao*LadC-LOV	CGGTGGCGGTGGCGGTGCCGCAATCATCTATGGCTTTGATAGC	CAGAATAACCTGTGCGGTCACATCAATCTGGGCTGC
pET GB1a *Ms*LadC-ccGGDEF	ACCGCACAGGTTATTCTG	GGCACCGCCACCGCCACCG
*Ms*LadC$^{\text{GCN4linker}}$	CAAAAACTATATGCTGGAGAACGAGGTGGCACGTCTGAAAAAACTGGTGAACAGCGATGCACTGACCGGTCTGC	GTTCTCCAGCATATAGTTTTTGCTCAGCAGCTCCTCCACTTTATCCTCCAGTGCGCTAACATCTTTCTGAATGCC
GCN4cc-*Ms*GGDEF	GTGGCGGTGCACTGGAGGATAAAGTGGAGGAGC	TGCACCGCCACCGCCACCGCCCTGAAAATA
*Ms*LadC-Q122L-N126V-K140V	CAGACCCTGCATGAAGAAAACCATCTGCTGAAAAGCAACG	TTCATGCAGGGTCTGAACCAGAATAACCAGTGCGCTAACAT
Ms-ccGGDEF	GGTGGCGGTGCACAGGTTATTCTGAATCAGACCCTGC	GCACCGCCACCGCCACCGCCCTGAAAATAAAGA
*Ms*LadC$^{\text{GB1-less}}$	CATCACCATCACCATCACGAGAATCTTTATTTTCAGGGCGGTGGC	GTGATGGTGATGGTGATGTTTCATGGTATATCTCCTTCTTAAAGTTAAACAAAATTATTTCTAG
*Ms*LadC-Q164E-R174A	CTGGTTATTCAGTGGAAACTGGCAAGCGCTCATATCAATACCATTACC	CCACTGAATAACCAGCTCATCTTCCAGAAAACGACGATTATGCAGAC
*Ms*LadC-N214E	TGTTTTATGCGTGGCAGCGATTTTGTTGCACGTTATGG	GCCACGCATAAAACATTCATTCAGGGTTTTTGCAATGGTACGCAG
*Ms*LadC-N214K	TGTTTTATGCGTGGCAGCGATTTTGTTGCACGTTATGG	GCCACGCATAAAACATTTATTCAGGGTTTTTGCAATGGTACGCAG
-N126V	GAGACCCTGCATGAAGAAAACCATCTGCTGAAAAGCAACAAAG	TTCATGCAGGGTCTCAACATTAGCAACCTG
*Ms*LadC-Y145Q-L146N	GTGAATATTGATGCACTGACCGGTCTGCATAATCGTC	TGCATCAATATTCACATTCTGTTCCAGCATCTCTTTGTTGCTTTTCAGC
*Ms*LadC^{GGAAF}	GCACGTTATGGTGGTGCAGCATTTACCATTCTGGCAATTGGTATGACCGA	ACCACCATAACGTGCAACAAAATCGCTGCCACGCATAAAACAATTATTC
*Ms*LadC-218-GMGG	GCAAAAACCCTGAATAATTGTTTTATGGGCATGGGCGGCTTTGTTG	CATAAAACAATTATTCAGGGTTTTTGCAATGGTACGCAGTGCTTCATC
*Ms*LadC-218-GGNH	GCAAAAACCCTGAATAATTGTTTTATGGGCGGCAACCATTTTGTTGCAC	CATAAAACAATTATTCAGGGTTTTTGCAATGGTACGCAGTGCTTCATC
*Ms*LadC-218-RAGG	GCAAAAACCCTGAATAATTGTTTTATGCGTGCAGGCGGTTTTGTTGC	CATAAAACAATTATTCAGGGTTTTTGCAATGGTACGCAGTGCTTCATC
*Ms*LadC-D221E	GCAAAAACCCTGAATAATTGTTTTATGCGTGGCAGCGAATTTGTTGC	CATAAAACAATTATTCAGGGTTTTTGCAATGGTACGCAGTGCTTCATC
-N126V-K140V	CGCACAGGTTATTCTGGTTCAGACCCTGCATGAAGAAAACC	CAGAATAACCTGTGCGCTAACATCTTTCTGAATGCCCAGGTAATG
*Ms*LadC-L125D	AATCAGACCCTGCATGAAGAAAACCATCTGCTGAAAAGCAAC	TCATGCAGGGTCTGATTATCAATAACCTGTGCGCTAACATCTTTCTGAA
*Ms*LadC-L125D-N126V-K140V	TTAGCGCACAGGTTATTGATGTTCAGACCCTGCATGAAGAAAAC	AATAACCTGTGCGCTAACATCTTTCTGAATGCCCAGGTAATGG
-E132L-N139L	CCATCTGCTGAAAAGCCTGAAAGAGATGCTGGAATATCTGGTGA	GCTTTTCAGCAGATGGTTCAGTTCATGCAGGGTCTGATTC

Table S2: Buffers for storage and biochemical characterization*.*

	**storage**	**UV/Vis spectroscopy**	***in vitro* DGC assay**
*Ms*LadC, its variants and *MsIs*	10 mM Tris/HCl, pH 8<br>50 mM NaCl<br>2 mM $MgCl_2$	10 mM Tris/HCl, pH 8<br>50 mM NaCl<br>2 mM $MgCl_2$	10 mM Tris/HCl, pH 8<br>50 mM NaCl<br>10 mM $MgCl_2$
MsIs variants	10 mM HEPES, pH 7<br>500 mM NaCl<br>2 mM $MgCl_2$	10 mM HEPES, pH 7<br>500 mM NaCl<br>2 mM $MgCl_2$	10 mM Tris/HCl, pH 8<br>50 mM NaCl<br>10 mM $MgCl_2$
AoMs and its variants	10 mM Tris/HCl, pH 8<br>50 mM NaCl<br>2 mM $MgCl_2$	10 mM Tris/HCl, pH 8<br>50 mM NaCl<br>2 mM $MgCl_2$	10 mM Tris/HCl, pH 8<br>50 mM NaCl<br>10 mM $MgCl_2$

Table S3: HDX data summary.

	Dark-state	Light-state
HDX reaction details	10 mM HEPES, 50 mM NaCl, 2 mM $MgCl_2$<br>pD = 7.0, 20 °C	10 mM HEPES, 50 mM NaCl, 2 mM $MgCl_2$<br>pD = 7.0, 20 °C
HDX time course (s)	10, 45, 180, 900, 3600	10, 45, 180, 900, 3600
HDX control samples	Unlabeled control (dark-state)	Unlabeled control (dark-state)
Back-exchange (mean / IQR)	not measured	
# of Peptides	94	92
Sequence coverage	89%	87%
Average peptide length / Redundancy	15 / 3.5	14 / 3.3
Replicates	3	3
Repeatability (average standard deviations for all time points – 10, 45, 180, 900, 3600 s, respectively)	0.13, 0.09, 0.12, 0.12, 0.18	0.17, 0.14, 0.10, 0.13, 0.33
Significant differences in HDX ($_{\Delta}$HDX > X D)	0.3 D	
